# Sensory Profile and Consumers’ Liking of Functional Ovine Cheese

**DOI:** 10.3390/foods4040665

**Published:** 2015-11-11

**Authors:** Antonella Santillo, Marzia Albenzio

**Affiliations:** Department of Agriculture, Food and Environmental Sciences (SAFE), University of Foggia, Via Napoli, 25, Foggia 71122, Italy; E-Mail: marzia.albenzio@unifg.it

**Keywords:** probiotic, ovine cheese, sensory profile, cheese liking

## Abstract

The present research was undertaken to evaluate the sensory profile and consumers’ liking of functional ovine cheese containing probiotic cultures. Ovine cheese was made from ewe’s milk by animals reared in extensive conditions; cheesemaking trials were performed by using rennet paste containing probiotic cells. Experimental cheeses were denoted: cheese manufactured using lamb rennet paste without probiotic (C), cheese manufactured using lamb rennet paste containing a mix of *Bifidobacterium lactis* and *Bifidobacterium longum* (BB), and cheese manufactured using lamb rennet paste containing *Lactobacillus acidophilus* (LA). Ovine cheese containing probiotic strains highlighted a more intense proteolysis and a greater level of short chain free fatty acids and conjugated linoleic acid due to the metabolic activity of the adjunct microflora. The sensorial profile of ovine cheese showed lower humidity and gumminess in cheeses containing probiotics as a consequence of differences in the maturing process; furthermore, probiotic cheeses scored higher ratings for salty and pungent attributes. An interaction effect of probiotic, gender, and age of the consumers was detected in the perceived and the expected liking. The higher rate of expected liking in all experimental cheeses is attributed to the information given, regarding not only the presence of probiotic strains but also the farming conditions and cheesemaking technology.

## 1. Introduction

Today, food is not merely viewed as a vehicle for essential nutrients to ensure proper growth and development, but as a route to optimal wellness. Functional foods are those which contain some health-promoting components that go beyond the traditional nutrients; one way in which foods can be modified to become functional is by adding probiotics [1]. The development of probiotic cheeses implies knowledge of the processing steps as well as a level of influence (positive or negative) on the survival of microorganisms, sensory acceptance, chemical stability, and microbiological conditions throughout shelf life [2].

The manufacture of probiotic cheese should have minimum changes when compared to traditional products [3] and must provide an adequate probiotic cell load upon cheese consumption. Cheeses could offer certain advantages as a delivery system of live probiotics to the gastrointestinal tract, having a higher pH than fermented milk and a high fat content that may protect the organisms during passage through the gastrointestinal tract [4]. The potential of different typologies of ovine cheese, e.g., semi-hard cheese and *pasta filata* stretched curd as a functional food delivering different probiotic bacteria has been previously reported [5,6,7]. Those reports showed that probiotics yielded a complex outfit of proteolytic and lipolytic enzymes able to influence not only cheese microbiology but also the maturing process. Furthermore, probiotics in ovine cheese were involved in the production of molecules such as essential amino acids [8], bioactive peptides [9], and polyunsaturated fatty acids and conjugated linoleic acid (CLA) [10].

The commercial success of any functional food, especially those containing probiotic strains, ultimately depends on taste, appearance, price, and health claim appeal to consumers [2]. Thus, in the development of probiotic ovine cheese, the sensory evaluation by consumers is a crucial and essential step that rules product innovation. Furthermore, the information about the characteristics of the innovative dairy product must be provided to the consumers, it being a factor able to increase consumers’ awareness and willingness to choose the new food. Napolitano *et al.* [11] reported that expectation induced by the information can affect the quality perception and oriented the consumers in their choice. The application of sensory methodology allows researchers and developers to obtain important results on the formulated product with respect to its descriptive sensory profile and acceptance on the consumer market [12]. Developing a sensory profile of the product helps to identify the principal sensory features of functional products, their negative and positive attributes compared to the conventional product. The results from a descriptive analysis test also provides a basis for determining those sensory attributes that are important to acceptance. Some authors [13,14] provided a very interesting guide for the sensory evaluation of ewe’s milk cheese with a trained panel. However, it is crucial to remember that a trained panel must not measure liking, acceptance, or preference. Once panelists are trained to identify and quantify attributes in products (or grades and defects as in product judging), they are no longer typical consumers [12]. In the light of these considerations, the aim of the present research was to study the sensory profile and consumer liking of functional ovine cheese containing probiotic cells.

## 2. Experimental Section

### 2.1. Lamb Rennet Paste and Ovine Cheese Production

Gentile di Puglia ewe’s milk was produced on a dairy farm located in the province of Foggia, Southern Italy; the experiment was conducted in early spring (March) when animals were reared in extensive conditions, fed mainly on fresh pasture with concentrate integration at the milking parlor. Rennet paste was produced according to the following protocol: briefly, abomasa were extracted from suckling lambs and the perivisceral fat was removed; the abomasa were stored in salt at 6 °C and 70% relative humidity for 3 months and then ground to obtain a paste.

The experimental rennet pastes used were: control lamb rennet paste (C), rennet paste containing *Lactobacillus acidophilus* (LA), and lamb rennet paste containing a mix of *Bifidobacterium lactis* and *Bifidobacterium longum* (BB). The ripened rennet paste was inoculated with fresh probiotic cells to obtain a final concentration of 10^9^ cfu/g of rennet one day before ovine cheese manufacturing. Three experimental cheesemaking trials were performed for each type of lamb rennet paste. Ewe’s milk from morning and afternoon milking was collected for two consecutive days, stored at 4 °C, and processed on the third day for cheese production. Three experimental cheesemaking trials were performed for each type of lamb rennet paste. Each cheesemaking trial was performed with the same batch of milk. Briefly, raw ewes’ milk was thermized (65 °C for 5 min) and then cooled to 38 °C. Rennet paste was added (30 mL of an aqueous solution 60% *w*/*v*) to obtain coagulation in 30 min, then the curd was cut to grain size. After molding and pressing, the curds were held at 42 °C for 5 h, then salted in brine for 12 h (20% *w*/*v* NaCl) and ripened for 45 day (12 °C; R.H. 90%). Cheese weight was about 1.5 kg. At 45 day of ripening, counts of probiotic bacteria in cheese were 7.4 × 10^7^ cfu/g and 7.1 × 10^7^ cfu/g of cheese for *L. acidophilus* and the mix of bifidobacteria, respectively. After 45 day of ripening, experimental cheeses were stored under vacuum at 4 °C.

### 2.2. Analyses on Cheese

#### 2.2.1. Determination of Proteolysis and Lipolysis

Total nitrogen (TN) and phosphotungstic acid-soluble nitrogen (PASN) were determined as described by Gripon *et al.* [15], and water-soluble nitrogen (WSN) was measured as described by Stadhouders [16]. The TN minus WSN gave the casein nitrogen [17]. The derivatized free amino acids (FAA) were separated, identified, and quantified by reversed phase-HPLC (Agilent 1260 Infinity, Santa Clara, CA, USA, equipped with a binary pump G1312A, automatic sampler G1313A, degassing system, and column oven thermostatized at 40 °C) on a Zorbax Eclipse AAA column (4.6 × 150 mm, 3.5 μm film thickness; Agilent PN9634000-9029). The mobile phases were (A) 40 nM NaH2PO4 (pH 7.8) and (B) acetonitrile:methanol:H2O (45:45:10, *v*/*v*/*v*). Quantization was done using the area under each peak with the Agilent software ChemStation (Agilent Technologies, Santa Clara, CA, USA). Detection was performed on an Agilent diode-array detector G1315B (Agilent Technologies, Santa Clara, CA, USA) and a fluorescence detector G1321A (Agilent Technologies, Santa Clara, CA, USA).

Total lipids from cheeses were extracted according to the method of de Jong and Badings [18]. Free fatty acid (FFA) derivatization was performed according to the method of Morrison and Smith [19]. FFA and CLA were separated on a capillary column (HP88; 100 m × 0.25 mm i.d., 0.20-μm film thickness; Agilent Technologies Inc. Santa Clara, CA, USA). The injector and flame ionization detector temperatures were 250 °C. The temperature was held at 70 °C for 4 min and then increased to 175 °C (13 °C/min, held for 27 min), then increased to a final temperature 215 °C (4 °C/min, held for 45 min). The split ratio was 1:20 and helium was the carrier gas, with a pressure of 227.5 kPa. Pure CLA isomers were purchased as FA methyl esters from Matreya Inc. (Pleasant Gap, PA, USA). All solvents were analytical grade from J. T. Baker Inc. (Milan, Italy). Analyses were performed in duplicate.

#### 2.2.2. Determination of Textural Parameters

Samples for cheese texture analysis were obtained by cutting a 1-cm-thick slice from the central diameter of the cheese wheel after 45 day of ripening. Then, 6 rectangular parallelepipeds, 1 × 1 cm thick and 2 cm long, were obtained from the slide. The cheese samples were left at 20 °C for 10 min before testing. Texture profile analysis was evaluated with an Instron 4301 tensile tester (Instron Ltd., High Wycombe, UK), using a modified compression device that avoids transversal elongation of the samples. Each sample underwent 2 cycles of 80% compression; force × time data were used to calculate the following parameters: hardness, cohesiveness, springiness, gumminess, and chewiness. A typical texture profile of cheese obtained using the Instron equipment is interpreted to obtain the parameters reported in the study. Hardness is the height of the second peak (H2) in the first bite; cohesiveness is the ratio of area on second bite to that of the first bite (A2/A1). Gumminess is the hardness × cohesiveness × 100 (A1 × (A2/A1) × 100); Elasticity is the difference between distance B and distance C (C is the same measurement made using an inelastic material); Chewiness is the hardness × cohesiveness × springiness (A1 × (A2/A1) × (B − C)).

#### 2.2.3. Sensory Analysis

Subjects were recruited among students and personnel of the University of Foggia. The consumer panel consisted of 80 subjects; the panel was homogeneous on the basis of age (20–45 years) and gender. In addition subjects were selected using predetermined screening criteria based on consumption of dairy products made from ewe’s milk and their awareness of probiotic food products; subjects were asked to give an example of a food containing probiotics.

The sensory analysis of ovine cheese was carried out on 45 day ripened cheese using the descriptive model of Coppola *et al.* [20] with a few modifications [21]. Before the sensory analysis, the untrained panel was preliminarily briefed on the use of the sensory attributes on a 9-point intensity scale (0–9) of the scorecard. The scorecard contained descriptive attributes according to Santillo *et al.* [22] adapted for ovine cheese, namely 2 for odor/flavor (overall intensity, acid, rancid), 8 for taste (overall intensity, salty, acid, pungent, bitter, sweet, mold, rancid), color (overall intensity, uniformity), appearance, and texture (overall uniformity, moisture, chalky, rubbery, grainy). Cheeses were taken out of the refrigerator 1 h before serving. Each sample was assigned a 3-digit random number, and cheese slices (1.5 mm thick) from the 3 replications of the same batch were mixed randomly so that all replications from the same batch were presented an equal number of times. A glass of water and unsalted crispy bread were provided and consumers were instructed to take a small bite of bread and a sip of water after each cheese tasting. Duplicate trays of samples were presented to the panel at 10-min intervals.

#### 2.2.4. Determination of Functional Ovine Cheese Liking

The perceived, expected, and actual liking was measured in accordance with the protocol proposed by Napolitano *et al.* [11]. The protocol provided for three sessions: in the first session subjects were asked to taste the ovine cheeses and rate their liking in blind conditions without receiving any information on the production protocol of cheese (perceived liking). In the second session the subjects received the information and were asked to rate their liking on the basis only of the information given (expected liking). In the third session consumers were given the cheeses along with the information and expressed their liking (actual liking). Consumers rated their liking in a 9-point hedonic scale anchored with “like extremely” and “dislike extremely” and with a neutral center point of “neither like nor dislike”.

In sessions 2 and 3 the following information concerning ovine cheese production was given to the consumers.

Ovine cheese—cheese made using milk from ewes reared in extensive conditions based on grazing on natural pasture. The production of cheese involved the use of whole milk heated to a temperature of 65 °C for few minutes and the use of rennet paste as coagulant. Rennet paste is a type of coagulant used for typical dairy productions; it is a white paste obtained from the abomasa of suckling lambs. The cheese is salted in brine and then subjected to aging at controlled temperature and humidity for up to 45 days.

Functional ovine cheese containing *Lactobacillus acidophilus*—cheese made using milk from ewes reared in extensive conditions based on grazing on natural pasture. The production of cheese involved the use of whole milk heated to a temperature of 65 °C for few minutes and the use of rennet paste as coagulant. Rennet paste is a type of coagulant used for typical dairy productions; it is a white paste obtained from the abomasa of suckling lambs. The cheese is salted in brine and then subjected to aging at controlled temperature and humidity for up to 45 days. The rennet paste contains live cells of *Lactobacillus* *acidophilus* recognized as probiotic able to exert beneficial effects on human health. The cheese contains high level of probiotic cells in accordance with the current guidelines for food products.

Functional ovine cheese containing bifidobacteria—cheese made using milk from ewes reared in extensive conditions based on grazing on natural pasture. The production of cheese involved the use of whole milk heated to a temperature of 65 °C for few minutes and the use of rennet paste as coagulant. Rennet paste is a type of coagulant used for typical dairy productions; it is a white paste obtained from the abomasa of suckling lambs. The cheese is salted in brine and then subjected to aging at controlled temperature and humidity for up to 45 days. The rennet paste contains live cells of *Bifidobacterium* *longum* and *Bifidobacterium lactis* recognized as probiotic able to exert beneficial effects on human health. The cheese contains high level of probiotic cells in accordance with the current guidelines for food products.

#### 2.2.5. Statistical Analysis

All the variables were tested for a normal distribution using the Shapiro-Wilk test [23]. Data were analyzed by ANOVA using the GLM procedure of SAS [24], and the effect of probiotic strain was tested on chemical and sensory attributes of ovine cheese. The effect of probiotic, age, and gender of the panelist, and the interaction of these variables, was studied for the perceived, expected and actual liking of the ovine cheese. When significant effects were found (*p* < 0.05), Student’s *t*-test was used to locate significant differences between means.

## 3. Results

Chemical composition of probiotic ovine cheese is presented in Table 1. Casein content at 45 day of ripening was lower in probiotic cheeses with respect to the control. PASN/TN and FAA showed the same behavior among experimental cheeses: the mentioned parameters were lower in control cheese, intermediate in LA and higher in BB cheese. Fat content was highest in BB cheese. Among the free fatty acids, butyric acid (C4:0) showed the highest levels in cheeses containing probiotics and total CLA was the highest in LA, intermediate in BB, and the lowest in control cheese.

**Table 1:** Chemical composition of probiotic ovine cheese at 45 day of ripening (*n* = 18).

Parameter	Cheese			SEM	Effect, *p*
	C	BB	LA		Probiotic
Casein, %	29.4 ^b^	23.8 ^a^	24.3 ^a^	0.4	**
PASN/TN, %	6.4 ^a^	10.9 ^c^	8.0 ^b^	0.5	*
FAA, μg/g cheese	3598.5 ^a^	7424.6 ^c^	6340.8 ^b^	169.9	**
Fat, %	26.7 ^a^	31.8 ^b^	29.9 ^a^	0.7	***
C 4:0, %	14.2 ^a^	24.1 ^b^	19.6 ^b^	1.2	*
CLA, %	0.9 ^a^	2.0 ^b^	2.3 ^c^	0.1	*

Cheese: C = cheese made with traditional lamb rennet paste; BB = cheese made with lamb rennet paste containing a mix of *B. longum* and *B. lactis*; LA = cheese made with lamb rennet paste containing *L. acidophilus*; SEM = standard error; * *p* < 0.05; ** *p* < 0.01; *** *p* < 0.001; ^a,b,c^ Mean values followed with different superscripts differ significantly; PASN = phosphotungstic acid-soluble nitrogen; TN = total nitrogen; FAA = free amino acids; CLA = conjugated linoleic acids.

**Table 2:** Rheological parameters of probiotic ovine cheese at 45 day of ripening (*n* = 18).

Parameter	Cheese			SEM	Effect, *p*
	C	BB	LA		Probiotic
Hardness, N	25.6	29.4	26.0	2.1	NS
Cohesiveness	0.1 ^a^	0.2 ^b^	0.2 ^a,b^	0.1	*
Gumminess, N	0.4 ^a^	0.9 ^b^	0.6 ^a,b^	0.1	*
Chewiness, Nxmm	4.5	5.8	4.5	1.0	NS
Elasticity, mm	7.9	7.9	7.9	0.8	NS

Cheese: C = cheese made with traditional lamb rennet paste; BB = cheese made with lamb rennet paste containing a mix of *B. longum* and *B. lactis*; LA = cheese made with lamb rennet paste containing *L. acidophilus*; SEM = standard error; * *p* < 0.05; NS = not significant; ^a,b^ Mean values followed with different superscripts differ significantly.

Textural parameters of experimental cheeses at 45 day of ripening are reported in Table 2. Cohesiveness and gumminess were affected by the addition of probiotic cells, being higher in BB cheese, intermediate in LA, and lower in C cheese.

Sensorial attributes of ovine cheese at 45 day of ripening are reported in Table 3. Among appearance attributes, differences emerged for humidity and gumminess, which turned out to be the lowest in both cheeses containing probiotics whereas graininess was the highest in LA cheese. No differences were reported for uniformity and chalky appearance. All the attributes ascribed to color and odor were not affected by the experimental treatment. Regarding taste attributes, salty scored the highest value in BB and pungent was higher in both probiotic cheeses. The overall taste intensity and acid, bitter, sweet, mold, and rancid attributes were not affected by treatment.

**Table 3:** Sensorial attributes of probiotic ovine cheese at 45 day of ripening.

Parameter		Cheese			SEM	Effect, *p*
		C	BB	LA		Probiotic
**Appearance**	Uniformity	5.1	5.4	5.1	0.3	NS
	Humidity	6.4 ^b^	4.8 ^a^	5.0 ^a^	0.3	***
	Chalky	3.8	4.0	3.7	0.4	NS
	Gumminess	5.1 ^b^	4.0 ^a^	3.8 ^a^	0.3	**
	Graininess	2.7 ^a^	2.8 ^a^	2.9 ^b^	0.3	NS
**Color**	Uniformity	6.3	5.9	5.8	0.2	NS
	Intensity	6.1	5.9	5.6	0.3	NS
**Odor**	Intensity	6.1	5.7	5.6	0.3	NS
	Acid	3.4	3.4	3.1	0.3	NS
	Rancid	1.8	1.7	1.8	0.3	NS
**Taste**	Intensity	5.9 ^a^	6.8 ^b^	6.1 ^a,b^	0.3	*
	Salty	4.3 ^a^	5.7 ^b^	4.8 ^a^	0.3	***
	Acid	2.7	3.3	3.2	0.3	NS
	Pungent	2.5 ^a^	4.6 ^b^	4.1 ^b^	0.3	***
	Bitter	2.7	2.5	2.8	0.3	NS
	Sweet	3.5	2.7	3.1	0.3	NS
	Mould	1.1	0.9	1.2	0.3	NS
	Rancid	1.0	1.3	1.9	0.4	NS

Cheese: C = cheese made with traditional lamb rennet paste; BB = cheese made with lamb rennet paste containing a mix of *B. longum* and *B. lactis*; LA = cheese made with lamb rennet paste containing *L. acidophilus*; SEM = standard error; * *p* < 0.05; ** *p* < 0.01; *** *p* < 0.001; NS = not significant; ^a,b^ Mean values followed with different superscripts differ significantly.

The rating given by the consumer panel during the three hedonic tests was not affected by the use of probiotic strains in ovine cheese production, scoring a mean value of 7.01 ± 0.2, 7.64 ± 0.25, and 7.34 ± 0.3 for the perceived, the expected, and the actual liking, respectively. The rating of ovine cheese liking is presented in Table 4. Cheese liking was not affected both by probiotic addition and consumers’ group, whereas an interaction effect of probiotic, gender, and age of consumers was detected in the perceived and the expected liking. A higher rate was expressed for the perceived liking of the BB and LA cheeses by female consumers over 30 years. On the contrary, females less than 30 years old scored lower rates for the probiotic cheeses. BB cheese received an intermediate score from males less than 30 years old, whereas LA cheese received intermediate scores from males over 30 years old. For expected liking, higher rates were found in female over 30 years for all the experimental cheeses, whereas LA cheese scored higher rates in males over 30 years. The actual liking of experimental cheeses was not influenced by age and gender. All experimental cheeses obtained a judgment from “moderately pleasant” to “very pleasant”.

**Table 4:** Rating of probiotic ovine cheese liking.

Parameter	Gender and Age	Cheese			SEM	Effect, *p*		
		C	BB	LA		Probiotic	Gender x Age	Probiotic × Gender × Age
Perceived liking	M < 30 (*n* = 19)	6.5 ^a^	7.1 ^a,b^	6.6 ^a^	0.2	NS	NS	*
	M > 30 (*n* = 21)	6.5 ^a^	6.5 ^a^	7.1 ^a,b^				
	F < 30 (*n* = 21)	7.1 ^a,b^	6.5 ^a^	6.6 ^a^				
	F > 30 (*n* = 19)	6.6 ^a^	7.3 ^b^	7.3 ^b^				
Expected liking	M < 30 (*n* = 19)	7.5 ^a^	7.3 ^a^	7.7 ^b^	0.2	NS	NS	*
	M > 30 (*n* = 21)	7.7 ^a^	7.6 ^a^	7.8 ^b^				
	F < 30 (*n* = 21)	7.5 ^a^	7.1 ^a^	7.1 ^a^				
	F > 30 (*n* = 19)	7.8 ^b^	8.2 ^b^	8.1 ^b^				
Actual liking	M < 30 (*n* = 19)	7.1	7.5	7.3	0.4	NS	NS	NS
	M > 30 (*n* = 21)	7.2	7.5	7.1				
	F < 30 (*n* = 21)	7.2	7.1	7.1				
	F > 30 (*n* = 19)	7.7	7.6	7.5				

Cheese: C = cheese made with traditional lamb rennet paste; BB = cheese made with lamb rennet paste containing a mix of *B. longum* and *B. lactis*; LA = cheese made with lamb rennet paste containing *L. acidophilus*; SEM = standard error; * *p* < 0.05; NS = not significant; ^a,b^ Mean values followed with different superscripts differ significantly.

## 4. Discussion

Cheese ripening involves different biochemical pathways, such as proteolysis and lipolysis ascribed to endogenous and exogenous factors, mainly enzymes yielded by rennet and microflora. Proteolysis is the main process in cheese ripening; determining changes in the texture due to the breakdown of the protein network, and in flavor formation through the release of peptides, free amino acids, and catabolic products [25]. Cheese containing a mix of *Bifidobacterium longum* and *Bifidobacterium* *lactis,* and cheese containing *Lactobacillus acidophilus* highlighted a more intense proteolysis than cheese without probiotic as evidenced by a greater hydrolysis of the intact casein fractions. The disruption of the casein matrix led to a major accumulation of nitrogen fractions comprising low molecular weight peptides and free amino acids. However, differences emerged among probiotic cheeses, with cheese containing bifidobacteria showing a higher proteolytic potential. Previous studies reported that ovine cheese containing a mix of *B. longum* and *B. lactis* showed greater caseins hydrolysis, evidencing that probiotic strains have a different impact on the proteolysis of cheese [4,25]. BB cheese showed a more complex FAA profile as an outcome of the proteinase and peptidase activities, evidencing the real contribution of adjunct microflora to cheese ripening [26,27]. Lipolysis is another important biochemical event that leads to the formation of FFAs which also contribute to cheese flavour with volatile compounds [28]. Lipases associated with lactic acid microflora are acknowledged to be selective for short chain fatty acid release [29]. The greater level of C4:0 in probiotic cheese could be attributed to the metabolic activity of the adjunct microflora; in particular, the ability of *L. acidophilus* to convert linoleic acid in to CLA is a consequence of the detoxification mechanism enacted by this probiotic strain. CLA enrichment led to an ameliorated composition of the fat fraction of ovine cheese; indeed the involvement of these polyunsaturated fatty acids has been reported in the prevention of many human diseases [30].

Cheese texture may be defined as a composite sensory attribute resulting from a combination of physical properties perceived by the senses of sight, touch, and hearing [31]. Cheese texture could be measured indirectly by using instrumental rheological techniques and is related to the composition, structure, and strength of the attractions between the structural elements of the cheese. The behavior of the texture profile revealed that cheeses containing bifidobacteria were less brittle, in accordance with Buriti *et al.* [32], probably as a consequence of the greater proteolysis associated with the cheese maturing process.

Evaluation of sensory attributes permits the definition of the perceived profile and overall acceptability by consumers of dairy products with innovative characteristics. The analysis of the sensorial profile of the cheeses permits the identification of specific attributes that could be preference drivers and the evaluation of the impact of health information on consumer preference, expectation, and choice. Higher scores for humidity and gumminess were in accordance with the minor proteolytic process observed in the control cheese; cheese without probiotic was judged as the cheese with an overall lower intensity for taste attributes. Regarding taste attributes, the score for the Pungent attribute doubled in probiotic cheese with respect to the control cheese as an outcome of the major accumulation of C4:0. Butyric acid is one the major odorants in Cheddar and Camembert [33], and is also an important component of Feta cheese, contributing greatly to its flavor and piquant taste [34]. The salty taste, together with the pungent attribute, led to higher overall intensity perceived upon consumption of cheese containing bifidobacteria compared to control cheese. It is worth noting that the drivers of cheese acceptability are different depending on the consumer’s gender; a previous study on Caciocavallo cheese reported that salty and piquant attributes were mostly enjoyed by male rather than female panelists [22].

Some food attributes are directly perceived as price and sensory properties; others must be communication objects as healthy and/or ethical aspects. Indeed, the perception of a healthier product (e.g., cheese containing high levels of probiotic microorganisms) could increase the actual acceptability of cheese [35]. The absence of differences among cheeses in the rating of ovine cheese liking is an encouraging result because the adjunct of probiotic cultures in cheese did not lead to the development of aroma and flavor defects that permit consumers to distinguish between a traditional and an innovative cheese. Overall, the manufacture of probiotic cheese should have minimum changes when compared to traditional products [2]. The addition of bifidobacteria in Gouda cheese [36] and in Cottage cheese [37] led to negative effects on cheese flavor, reducing the acceptability of probiotic cheese with respect to a traditional one. In Cheddar cheese, Ong *et al.* [38] reported that cheese acceptance depended on the probiotic microorganism used in cheesemaking, evidencing the advisability of consumer acceptance testing whenever selected probiotic strains are used in the cheesemaking process.

The perceived liking of ovine cheese determined in blind conditions represented the baseline for the evaluation of the impact of information on consumers’ expectation; in particular, grouping the panel on the base of gender and age allowed for the evaluation of information on different segments of consumers. The expected liking was different from the perceived liking expressed in blind conditions, indicating that a negative disconfirmation occurred: higher rates of expected liking highlighted that the products are worse than expected although the overall rate referred to an acceptable product with a score over 6.5. In general, the higher rates of expected liking in all experimental cheeses was attributed to the information given to consumers concerning not only the presence of probiotic strains in cheese but also the farming conditions of the ewes, and the particular cheesemaking technology. It can be argued that consumers are prone to consider the farming conditions and the production of ovine cheese using traditional protocols as an added value to the quality of ovine cheese. Napolitano *et al.* [11] reported that information about organic farming can be a major determinant of cheese liking when comparing organic and conventional cheese. When the cluster of females over 30 years was considered, a higher weight was ascribed to the information related to the healthy effect of cheese when compared to male consumers and females less than 30 years of age. The higher expected liking for LA cheese in males over 30 years old could be ascribed to the fact that *L. acidophilus* is one of the most commonly used probiotic strains used in dairy foods. Probiotic products seemed to create a higher expectation in females over 30 years, probably due to an elevated consciousness of the benefits of healthy food. Indeed, a choice experiment on semi-hard cheese highlighted that female, rather than male participants, were affected by health information on their diet choices, evidencing clear diet-health awareness [39]. When actual liking was measured, scores moved towards the expectation liking rate, evidencing that assimilation of the information given contributed to the increase of ovine cheese liking.

Probiotic cheese combines nutritional and functional properties and is promising for the expansion of dairy products from ovine milk. The experimental cheeses containing probiotics were judged as the cheeses with an overall higher intensity for taste attributes in accordance with the more intense proteolysis and a greater level of short chain free fatty acids and conjugated linoleic acid. The results from the present research also highlight that the design and development of functional ovine cheese represents a technological innovation that needs to be supplied with adequate information in order to be able to orient consumers in their food choice. Nowadays, consumers are aware of the great impact of nutrition on health and reward ovine cheese as a food product associated with a sustainable production system. Special attention must be paid to the sensory profile of probiotic ovine cheese in order to provide information on the cheese attributes able to influence consumers’ liking.
